# A qualitative transcriptional signature for predicting microsatellite instability status of right-sided Colon Cancer

**DOI:** 10.1186/s12864-019-6129-8

**Published:** 2019-10-23

**Authors:** Yelin Fu, Lishuang Qi, Wenbing Guo, Liangliang Jin, Kai Song, Tianyi You, Shuobo Zhang, Yunyan Gu, Wenyuan Zhao, Zheng Guo

**Affiliations:** 1 Department of Systems Biology, College of Bioinformatics Science and Technology, Harbin Medical University, Harbin, 150086 China; 2 Department of Bioinformatics, Key Laboratory of Ministry of Education for Gastrointestinal Cancer, School of Basic Medical Sciences, Fujian Medical University, Fuzhou, 350122 China; 3 Key Laboratory of Medical Bioinformatics, Fujian Province, Fuzhou, 350122 China

**Keywords:** Right-sided colon cancer, Microsatellite instability status, Gene expression profiles, Relative gene expression orderings, Qualitative transcriptional signature

## Abstract

**Background:**

Microsatellite instability (MSI) accounts for about 15% of colorectal cancer and is associated with prognosis. Today, MSI is usually detected by polymerase chain reaction amplification of specific microsatellite markers. However, the instability is identified by comparing the length of microsatellite repeats in tumor and normal samples. In this work, we developed a qualitative transcriptional signature to individually predict MSI status for right-sided colon cancer (RCC) based on tumor samples.

**Results:**

Using RCC samples, based on the relative expression orderings (REOs) of gene pairs, we extracted a signature consisting of 10 gene pairs (10-GPS) to predict MSI status for RCC through a feature selection process. A sample is predicted as MSI when the gene expression orderings of at least 7 gene pairs vote for MSI; otherwise the microsatellite stability (MSS). The classification performance reached the largest F-score in the training dataset. This signature was verified in four independent datasets of RCCs with the F-scores of 1, 0.9630, 0.9412 and 0.8798, respectively. Additionally, the hierarchical clustering analyses and molecular features also supported the correctness of the reclassifications of the MSI status by 10-GPS.

**Conclusions:**

The qualitative transcriptional signature can be used to classify MSI status of RCC samples at the individualized level.

## Background

Microsatellite instability (MSI), the insertion or deletion mutations in microsatellites [1], is a molecular hallmark of a deficient mismatch repair (dMMR) system and accounts for about 15% of colorectal cancer (CRC) [2]. Results from some studies seem that the MSI feature is associated with good prognosis, and the stage II and III CRC patients with MSI cannot benefit from 5-fluorouracil (5-Fu)-based adjutant chemotherapy (ACT) [3–5], which is regarded as the standard treatment for stage II and III CRC patients after surgery. So, a precise classification is needed to aid appropriate decisions on 5-Fu-based ACT treatment of patients.

Today, the most common method to test MSI status is polymerase chain reaction (PCR) amplification analysis of specific microsatellite repeats, which is considered as the ‘golden standard’ method [1, 6]. However, the PCR technology exists high measurement variations between different laboratories [7, 8], which are mainly due to the effects of the tumor cell percentage and DNA degradation during sample storage and preparation [8–11]. Additionally, the presence of instability is defined by comparing the length of microsatellite repeats in the tumor sample and the normal sample [1, 6, 12–14]. MSI can also be detected by immunohistochemistry (IHC). But IHC method only provides a semi-quantitative evaluation of the expression levels of the four MMR proteins (MLH1, MSH2, MSH6 and PMS2) [2], and also exists high measurement variations between different laboratories [15–17], which are primarily due to the effects of sample preprocessing, such as fixation of tissues, detection reagents and selection of antibody [16, 17]. Moreover, since the results of IHC can be greatly affected by the interpretation of the specificity of staining, when the levels of the MMR proteins analyzed are low, good performance of IHC requires highly skilled personnel and pathologist’s experience in interpretation [14, 15]. Therefore, the traditional PCR and IHC methods both have some limitations in determining the MSI status of CRC.

Recently, many alternative methods based on tumor genomic data via next-generation sequencing (NGS) panels have been developed to determine MSI status of patients [1, 18, 19]. For instance, Vanderwalde et al. used a NGS panel comprised of 592 genes to determine MSI status [1]. However, NGS currently has been limited to some highly specialized laboratories [20]. Besides, there has no consensus about NGS gene panels to determine MSI status and each laboratory determine the appropriate mutation load threshold based on its NGS gene panel and technique [1, 18–20]. What’s more, the use of the NGS-based methods that require DNA extraction often leads to false-negative or uncertain results in challenging tumor samples due to the tumor DNA dilution and the percentage of tumor cells within a sample [20, 21]. Another method based on gene expression measurement also has been developed to identify MSI status of CRCs [6], which, however, is sensitive to the systematic inter-laboratory biases especially batch effects of microarray and RNA-sequencing experiments [22]. In general, quantitative transcriptional signatures based on absolute expression values is sensitive to the batch effects and thus lack robustness for clinical applications [23]. In contrast, the type of qualitative transcriptional signatures based on the within-sample relative expression orderings (REOs) of genes have strong robustness against the experimental batch effects and can be applied to individual samples directly [24]. Besides, we have demonstrated that they are rather robust against the proportions of tumor epithelial cell variations in tumor tissues sampled from different tumor locations [25], amplification bias for minimum samples [26], and partial RNA degradation during sample preparation [27]. For example, we have reported that more than 90% of the REOs of gene pairs in the fresh-frozen samples are maintained in their paired formalin-fixed paraffin-embedded samples and largely unaffected by the storage time [27], indicating that the vast majority of the REOs of gene pairs are rather robust. Thus, the gene pairs of signature were less vulnerable to degradation. Therefore, it is worthwhile to apply the within-sample REOs to find robust qualitative transcriptional signatures.

Colorectal cancers deriving from proximal or distal of splenic flexure are classified as right-sided or left-sided colon cancer (RCC or LCC), respectively [28]. Consistent with the differences in anatomy location, RCC and LCC have unique gene expression characteristics, different molecular pathways of carcinogenesis [28, 29] and different clinical features [30]. Therefore, it would be necessary to develop signatures to predict MSI status for RCC and LCC, respectively. Because of the high incidence of MSI in the RCC, we developed a REOs-based qualitative signature for predicting MSI status of RCC patients in this work, which was validated in independent datasets.

## Results

### Identification and validation of the signature for MSI status of RCC

**Fig. 1:**
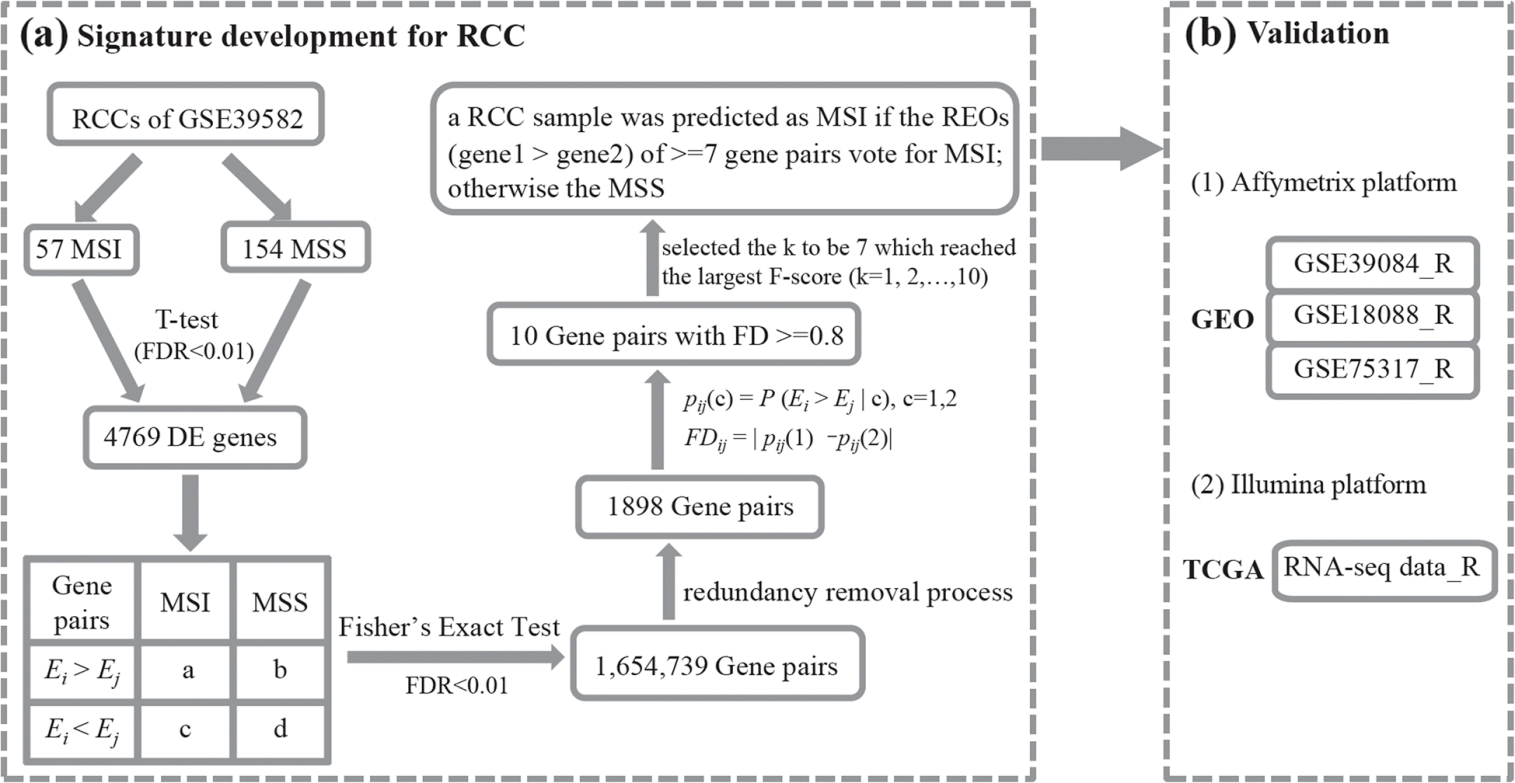
The flowchart of this study, as exemplified by the development and validation of predicting MSI status signature for patients with RCC (see Methods)

**Fig. 2:**
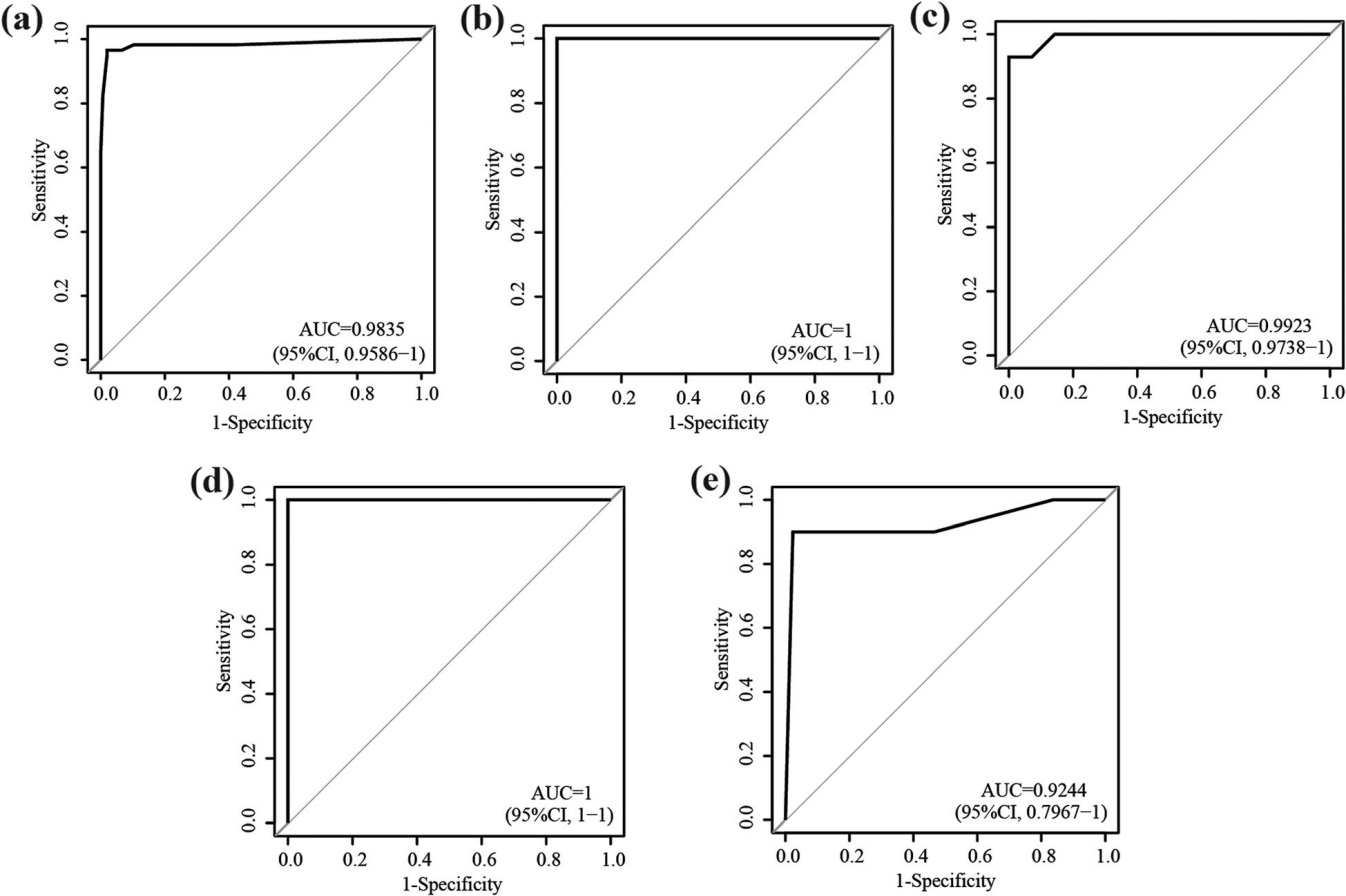
The ROC curves for 10-GPS in four datasets. **a** the training dataset. **b** the RCCs of GSE39084. **c** the RCCs of GSE18088. **d** the RCCs of GSE75317. **e** the RCCs of TCGA

The GSE39582 with the largest samples of RCC, including 57 MSI and 154 MSS was used as the training data for extracting a REOs-based signature. Firstly, we identified 4769 MSI-related differentially expressed (DE) genes (Student’s *t*-test, FDR < 0.01, Additional file 1: Table S1) between the 57 MSI RCC samples and the 154 MSS RCC samples. From all the gene pairs formed by these DE genes, we identified 1,654,739 gene pairs, whose specific REO pattern occurred more frequently in the MSI samples than in the MSS samples (Fisher’s exact test, FDR < 0.01). The larger FD of a REO pattern, the stronger classified ability of the REO pattern can classify the status of MSI. We further narrowed down the number of gene pairs to 1898 through a redundancy removal process by keeping only one with the largest FD value of those gene pairs sharing a common gene (see Methods, Fig. 1a). From these gene pairs, we extracted 10 gene pairs with the FD at least 0.8. These 10 gene pairs were used as the signature for predicting MSI status of RCC, denoted as 10-GPS (Table 1). A RCC sample was predicted as MSI if the REOs of at least seven gene pairs in the 10-GPS vote for MSI; otherwise the MSS. According to the classification rule, the F-score of the signature in the training data was 0.9727, with a sensitivity of 0.9649 and a specificity of 0.9805. The area under the curve (AUC) of the receiver operating characteristic (ROC) curve was 0.9838 (Fig. 2a).

**Table 1:** The Composition of 10-GPS

signature	gene1	gene2	signature	gene1	gene2
pair1	*HNRNPL*	*CDC16*	pair6	*STRN3*	*TMEM192*
pair2	*MTA2*	*VGF*	pair7	*HPSE*	*BCAS3*
pair3	*CALR*	*SEC22B*	pair8	*PRPF39*	*ATF6*
pair4	*RASL11A*	*CAB39L*	pair9	*CCRN4L*	*GRM8*
pair5	*LYG1*	*DHRS12*	pair10	*AMFR*	*DUSP18*

Notes: A RCC sample was classified as MSI if the REOs (gene1 > gene2) of at least 7 of the gene pairs in the 10-GPS vote for MSI; otherwise the MSS

We tested the 10-GPS in four independent cohorts of RCC samples (Fig. 1b), the F-scores of the classification by 10-GPS were 1, 0.9630, 0.9412 and 0.8798, respectively, as shown in the Table 2, and the AUCs were 1, 0.9923, 1 and 0.9244, respectively (Fig. 2b, c, d and e).

**Table 2:** The performances of the 10-GPS in RCCs of the independent datasets

	pre-MSI^a^ (MSI:MSS)^b^	pre-MSS^a^ (MSI:MSS)^b^	sensitivity	specificity	F-score
GSE39084_R	13 (13:0)	18 (0:18)	1	1	1
GSE18088_R	13 (13:0)	15 (1:14)	0.9286	1	0.9630
GSE75317_R	8 (8:0)	18 (1:17)	0.8889	1	0.9412
TCGA_R	15 (9:6)	38 (1:37)	0.900	0.8605	0.8798
Total_RCCs	49 (43:6)	89 (3:86)	0.9348	0.9348	0.9348

Notes: ^a^ represents the predicted MSI status by 10-GPS; ^b^ represents the original MSI status; GSE_R represents the RCC samples; Total_RCCs represents all the samples of RCC

### Transcriptome assessment of the signature-disconfirmed RCC samples

**Fig. 3:**
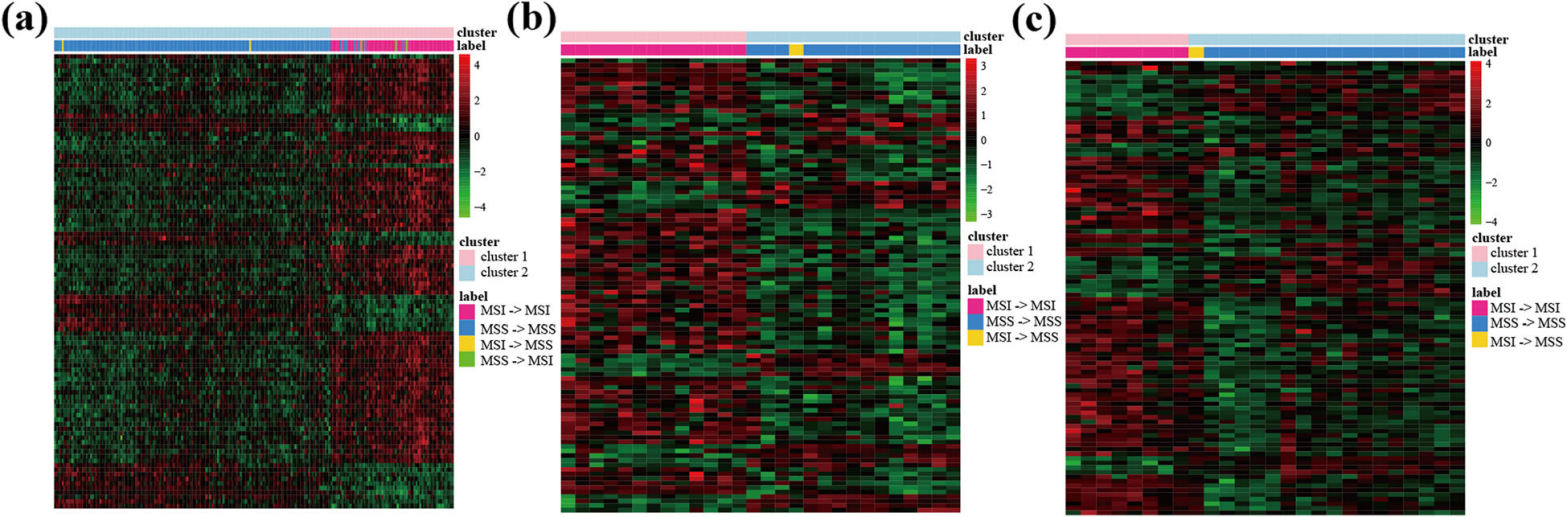
The complete linkage hierarchical clustering of the RCC samples in the (**a**), training dataset, (**b**) GSE18088 and (**c**) GSE75317 based on the differentially expressed genes between the signature-confirmed MSI and MSS samples. X- > Y, X represents the original MSI status and Y represents the reclassified MSI status by 10-GPS

In the training data, there were a total of five signature-disconfirmed samples. We compared the gene expression patterns of the five signature-disconfirmed samples with the 206 signature-confirmed samples through clustering analysis. Firstly, we identified 5664 DE genes (Student’s *t*-test, FDR < 0.01) between the 55 signature-confirmed MSI and the 151 signature-confirmed MSS samples. Secondly, using the expression levels of the top 100 significant DE genes, the samples were divided into two subgroups using the complete linkage hierarchical clustering based on the Euclidean distance (Fig. 3a). The results showed that all of the two MSI samples reclassified as MSS by the 10-GPS were clustered with the signature-confirmed MSS samples, and all of the three MSS samples reclassified as MSI were clustered with the signature-confirmed MSI samples.

Similarly in the two of the four validation datasets of RCC (GSE18088 and GSE75317), all of these two MSI samples reclassified as MSS by our signature were clustered with the corresponding signature-confirmed MSS samples (Fig. 3b and c), respectively. These results provided transcriptional evidence of the correctness of the prediction of 10-GPS.

### Genome assessment of the signature-disconfirmed RCC samples

It is known that BRAF^V600E^ mutations and CpG island methylator phenotype (CIMP)-positive frequently occur in MSI CRCs, whereas KRAS mutations (in codons 12 or 13) frequently occur in MSS CRCs [2, 31]. In the training data, for the three MSS samples which were reclassified as MSI by 10-GPS, two patients were KRAS wild-type, BRAF mutant and CIMP-positive and one patient was KRAS wild-type. For the two MSI samples which were reclassified as MSS, one was BRAF wild-type and CIMP-negative, as shown in Table 3.

**Table 3:** The molecular characteristics of the five signature-disconfirmed RCC samples in the training dataset

original_MSI. status	predicted_MSI. status	KRAS. status	BRAF. status	CIMP. status
MSS	MSI	wild type	NA	NA
MSS	MSI	wild type	mutation	+
MSS	MSI	wild type	mutation	+
MSI	MSS	wild type	mutation	+
MSI	MSS	NA	wild type	–

In the TCGA validation dataset of RCC, there were seven signature-disconfirmed samples. Because mutation of MMR genes can result in MSI [32], we observed the mutation status of the MMR genes in the signature-disconfirmed samples. There were only five samples with mutation data. And two of the four MSS samples which were reclassified as MSI by 10-GPS were MSH6 mutant (Additional file 2: Table S1). These results supported that MSI status of these samples reclassified by 10-GPS might be reliable.

### Prognosis assessment of the signature-disconfirmed RCC samples

Then, we also evaluated the reliability of the reclassifications by 10-GPS through survival analyses based on the knowledge that stage III MSI CRCs treated with surgery only have better prognoses than MSS CRCs [2] and that stage III MSS CRC patients treated with 5-Fu-based ACT after surgery have improved outcomes than patients treated with surgery only [3–5]. And the survival benefit of ACT was only observed in stage III patients [3, 4]. In the 32 stage III RCC samples of the training data for patients treated with surgery only, one of the 19 MSS sample was reclassified as MSI and one of 13 MSI sample was reclassified as MSS by the 10-GPS. In the 54 (46 MSS and 8 MSI) stage III RCC samples for patients receiving 5-Fu-based ACT, the original MSI status of all samples were confirmed by the 10-GPS. By comparison, the MSS patient reclassified as MSI had longer RFS (130 months) than the MSI patient reclassified as MSS (31 months) by the signature. The survival difference between patients with predicted MSI status by 10-GPS were more significant than the difference between patients with the original MSI status due to the two reclassified samples (Additional file 3).

### Identification and validation of the signature for MSI status of LCC

We applied the 10-GPS to LCC samples and CRC samples without clear location information (Additional file 2: Table S2). The results showed that the performance was reduced when applying the 10-GPS to predict MSI status of LCC. Therefore, we also tried to develop a signature to identify MSI status of the LCC patients in the same way as in RCC. Eventually, these six gene pairs were used as the signature for predicting MSI status of LCC, denoted as 6-GPS (Additional file 2: Table S3). A LCC sample was predicted as MSI if the REOs of at least four gene pairs in the 6-GPS vote for MSI; otherwise the MSS. According to the classification rule, the F-score of the signature in the LCC training data was 0.9983, with a sensitivity of 1 and a specificity of 0.9966. And also, the 6-GPS was well validated in four independent cohorts of LCC samples (Additional file 2: Table S4).

## Discussion

We developed qualitative transcriptional signatures consisting of 10 and 6 gene pairs to robustly predict MSI status of RCC and LCC at individualized level, which were validated in four independent datasets. Notably, the hierarchical clustering analyses and molecular characteristics supported the correctness of the reclassifications of the MSI status by our signature for some samples whose MSI statuses were determined by the PCR testing. Besides, using gene pairs with large FD (see Methods) of a REO pattern between MSI and MSS samples, we can exclude gene pairs affected by various factors such as RNA degradation and tumor cell percentage and obtain a classifier with high predictive performance of MSI status. Thus, it is possible for our signature to identify the MSI status of CRC samples, which could not be determined by traditional standard methods.

In this study, we selected MSI-related gene pairs formed by the DE genes identified between MSI and MSS RCC samples. Some DE genes are known to be associated with microsatellite instability, prognosis and metastasis of CRC. For example, *CAB39L* have mononucleotide repeats in the coding regions that could be targets for frameshift mutation in CRC with microsatellite instability [33]. Another gene, *MTA2*, is one of metastasis-associated tumor gene family members and was an important prognosis biomarker of CRC [34]. Besides, it is reported that overexpression of *AMFR* is significantly related to poor survival for CRC [35]. Additionally, the differential expression of these genes could cause reversal REOs of the selected gene pairs between MSI and MSS samples, and thus these gene pairs could have the ability to classify the MSI status.

In the process of screening MSI-related gene pairs of RCC, we extracted gene pairs by adjusting different FD thresholds. Then, it was found that when FD > 0.9, there were no gene pairs remaining. When FD > 0.7, there were 65 gene pairs were extracted. The classification performance reached the largest F-score (0.9630) of sensitivity (0.9649) and specificity (0.9610) in the training data according to the following decision rule: a sample is predicted as MSI when the REOs of at least 39 gene pairs vote for MSI; otherwise the MSS. Compared with the result of FD > 0.8, the performance of classification was slightly worse and it had more gene pairs. So we chose 0.8 as the threshold for FD. Similarly, based on the same considerations, we chose 0.9 as the threshold for FD in the process of screening MSI-related gene pairs of LCC.

The REO-based method was first proposed by Donald Geman et al. in 2004 [36]. The method has been proposed as a simple, accurate and easily interpretable decision rule for classification of gene expression profiles [37]. What’s more, it is robust against the experimental batch effects and avoid the need of inter-sample data normalization and can be applied at individualized level [23, 24, 36]. So, there were many studies by others and us developing several prognostic and predictive biomarkers based on this method for different cancers [38–51]. It indicated that the clinical applicability of the signatures based on the robust qualitative REO information extracted from the quantitative measurements of gene expression, rather than the “exact” quantitative measurements themselves [52]. Given cost considerations and the often-limited quantity of tumor material available for testing in many cancer patients, NGS-based tumor profiling, which provides the basis for the concept of “a sequence for all” [53]. So, we have been focusing on developing qualitative transcriptional signatures to form the “a sequence for all” for CRC. All these signatures can be assessed in a single NGS assay, facilitating the optimum treatment of stage II-III CRC patients. In summary, we developed qualitative signatures for predicting MSI status of RCC and LCC, as a part of “a sequence for all” for CRC.

## Conclusions

Currently, common methods for detecting MSI status of CRC such as PCR and IHC-based methods, exist high measurement variations between different laboratories, which have limited clinical utility. Herein, we developed robust qualitative transcriptional signatures to classify MSI status of RCC and LCC at the individualized level, as a part of “a sequence for all” for CRC. The simplicity and robustness of the signature would make it convenient in clinical settings and worthy to further validate in a prospective clinical trial.

## Methods

### Data sources and data preprocessing

The gene expression datasets used in this study were downloaded from the Gene Expression Omnibus (GEO, http://www.ncbi.nlm.nih.gov/geo/) database and The Cancer Genome Atlas data portal (TCGA, http://cancergenome.nih.gov/) (Table 4). As MSI-L patients are usually treated in a way similar to MSS patients in clinical practice, it is reasonable to group MSI-L with MSS [1]. In this study, we grouped MSI-L with MSS. The training data for extracting a REOs-based signature of RCC was GSE39582, including 57 MSI and 154 MSS of 211 RCCs. The GSE39582 dataset recording survival information of patients were used as the test for survival analyses.

**Table 4:** The datasets analyzed in this study from GEO and TCGA

	GSE39582 (*n* = 566)	GSE39084 (*n* = 70)	GSE18088 (*n* = 53)	GSE75317 (*n* = 59)	GSE13067 (*n* = 74)	GSE13294 (*n* = 155)	TCGA (*n* = 457)
Stage							
I	33	8	–	6	–	–	75
II	264	23	53	24	–	–	178
III	205	16	–	17	–	–	130
IV	60	22	–	12	–	–	64
Microsatellite status							
MSI	75	16	19	11	11	78	11
MSS	444	54	34	48	63	77	81
Location							
Right	224	31	28	26	–	–	261
Left	342	30	25	33	–	–	177
MSI_proportion							
Right	57:154^a^ (27.0%)^b^	13:18 (41.9%)	14:14 (50.0%)	9:17 (34.6%)	–	–	10:43 (18.9%)
Left	18:290^c^ (5.8%)^d^	3:27 (10.0%)	5:20 (20.0%)	2:31 (6.1%)	–	–	1:33 (2.9%)
MSI detection	PCR	PCR or IHC	PCR	PCR	PCR	PCR	PCR
Adjuvant chemotherapy							
Yes	233	–	–	–	–	–	–
No	316	–	53	–	–	–	–

Notes: The data from GEO were produced by the same gene expression profiling platform (GPL570, Affy-HG-U133_Plus_2). a represents the number of MSI and MSS of RCCs, respectively; b represents the proportion of MSI in RCCs; c represents the number of MSI and MSS of LCCs, respectively; d represents the proportion of MSI in LCCs

The Robust Multi-array Average algorithm [54] was used for preprocessing the raw data measured by the Affymetrix platform. Using the corresponding platform files, probes were mapped to genes. For each sample, the expression measurements of several probes mapping to a gene were averaged to obtain a single measurement. Probes were discarded if they did not match any gene or matched multiple genes. The RNA-seq expression data were downloaded from the Broad Firehose webpage (http://gdac.broadinstitute.org/). For RNA-seq data derived from Illumina HiSeq 2000 RNA Sequencing Version 2, we directly downloaded the RSEM-normalized format and log2-transformed. We also downloaded the somatic mutation data of CRC from the Broad Firehose webpage.

Currently, most of the CRC data we collected were microarray datasets. With the development of the NGS technology, the RNA-seq data are increasing. In order to apply our signature to RNA-seq data, we used the expression data of overlapping Gene IDs of Affymetrix and Illumina platforms.

### Signature development for predicting MSI status of RCC

We identified MSI-related gene pairs from the DE genes between MSI and MSS samples. For a gene pair, *i* and *j*, with expression values of *E*_*i*_ and *E*_*j*_, whether the frequency of a specific REO pattern (*E*_*i*_ *> E*_*j*_ or *E*_*i*_ *< E*_*j*_) was significantly higher in the MSI samples than the frequency in the MSS samples was evaluated by Fisher’s exact test [55]. The MSI-related gene pairs detected with FDR < 0.01. Then, the frequency difference (FD) was calculated for each MSI-related gene pair between the two groups (Formula 1). The larger FD of a REO pattern, the stronger classified ability of the REO pattern can classify the status of MSI and avoid the effect of degradation. It is more likely to be applied to multiple data sources produced by different laboratories. So, considering that some genes are influenced by RNA degradation, for a gene which appeared in multiple gene pairs, we kept only the gene pair with the largest FD value and discarded others.

*p*_*ij*_ (*c*) = *P* (*E*_*i*_ > *E*_*j*_ | *c*), c = 1,2, the probabilities of observing *E*_*i*_ > *E*_*j*_ in each class.

*FD*_*ij*_ = *p*_*ij*_ (1) − *p*_*ij*_ (2), the FD value of gene pair (*i*, *j*) [Formula 1].

After that, the gene pairs with the FD value at least 0.8 were identified as the signature to predict MSI status of RCC. A sample was labeled as MSI if the REOs of at least *k* gene pairs in the signature are consistent with the specific patterns (*E*_*i*_ > *E*_*j*_) of the training samples, and vice versa. For each *k* ranging from one to the number of gene pairs in the signature, we could compute a F-score. Finally, we selected the *k* which could reach the largest F-score.

2 $$ F- score=2\times sensitivity\times specificity\div \left( sensitivity+ specificity\right) $$

### Sample clustering

Student’s *t*-test was performed to identify DE genes between the MSI and MSS patients confirmed with the original MSI status by the signature. Complete linkage hierarchical clustering was performed to stratify CRC samples into subgroups. The similarity between samples was evaluated by the Euclidean distance based on the expression measurements of DE genes.

### Statistical analyses

For microarray data, we selected DE genes between two classes of samples using Student’s *t*-test. The MSI rate in different groups was evaluated by Fisher’s exact test. The Benjamini-Hochberg procedure for multiple testing was used to adjust *p* values in order to control the false discovery rate (FDR) [56]. Log-rank test was used to assess the difference between the Kaplan-Meier estimates of the RFS in the two different groups [57]. Hazard ratios (HRs) and 95% confidence intervals (CIs) were generated using the univariate Cox proportional hazards model. All statistical analyses were performed using the R 3.5.1 (http://www.r-project.org/).

## Supplementary information

**Additional file 1: Table S1.** The DE genes (FDR<0.01) between the 57 MSI RCC samples and the 154 MSS RCC samples. **Table S2.** The DE genes (FDR<0.01) between the 18 MSI LCC samples and the 290 MSS LCC samples.(XLS 692 kb)

**Additional file 2: Table S1.** The mutation status of the four MMR genes of the five signature-disconfirmed RCC in the TCGA dataset. **Table S2.** The performances of the 10-GPS in LCCs and CRCs of the independent datasets. **Table S3.** The Composition of 6-GPS. **Table S4.** The performances of the 6-GPS in LCCs of the independent datasets.(DOCX 18 kb)

**Additional file 3.** The Kaplan-Meier survival curve for the prediction of 10-GPS and original MSI status, respectively. (TIF 524 kb)

## Data Availability

All data analyzed in this study were downloaded from the public database: GEO and TCGA.

## Abbreviations

5-Fu: 5-fluorouracil
ACT: adjutant chemotherapy
AUC: area under the curve
CIMP: CpG island methylator phenotype
CRC: colorectal cancer
DE: differentially expressed
dMMR: deficient mismatch repair
FD: frequency difference
FDR: false discovery rate
GPS: gene pairs
IHC: immunohistochemical
LCC: left-sided colon cancer
MSI: microsatellite instability
MSS: microsatellite stability
NGS: next-generation sequencing
PCR: polymerase chain reaction
RCC: right-sided colon cancer
REOs: relative expression orderings
ROC: receiver operating characteristic
