# Supplementary material for: A qualitative transcriptional signature for predicting microsatellite instability status of right-sided Colon Cancer
Source: BMC Genomics. 2019 Oct 23;20:769. doi: 10.1186/s12864-019-6129-8 (PMC6813057; doi:10.1186/s12864-019-6129-8)
Supplement: Supplementary file 2 — Additional file 2: Table S1. The mutation status of the four MMR genes of the five signature-disconfirmed RCC in the TCGA dataset. Table S2. The performances of the 10-GPS in LCCs and CRCs of the independent datasets. Table S3. The Composition of 6-GPS. Table S4. The performances of the 6-GPS in LCCs of the independent datasets.(DOCX 18 kb) [file 12864_2019_6129_MOESM2_ESM.docx]

**Supplementary Table**

**Table S1. The mutation status of the four MMR genes of the five signature-disconfirmed RCC in the TCGA dataset.**

| **original_MSI.**  **status** | **predicted_MSI.**  **status** | **MLH1.**  **status** | **MSH2.**  **status** | **MSH6.**  **status** | **PMS2.**  **status** |
| --- | --- | --- | --- | --- | --- |
| MSS | MSI | wild type | wild type | mutation | wild type |
| MSS | MSI | wild type | wild type | mutation | wild type |
| MSS | MSI | wild type | wild type | wild type | wild type |
| MSS | MSI | wild type | wild type | wild type | wild type |
| MSI | MSS | mutation | wild type | wild type | wild type |

**Table S2. The performances of the 10-GPS in LCCs and CRCs of the independent datasets.**

|  | **pre-MSI^a^**  **(MSI:MSS)**^b^ | **pre-MSS**^a^  **(MSI:MSS)**^b^ | **sensitivity** | **specificity** | **F-score** |
| --- | --- | --- | --- | --- | --- |
| **GSE39084_L** | 3(3:0) | 27(0:27) | 1 | 1 | 1 |
| **GSE18088_L** | 4(4:0) | 21(1:20) | 0.8 | 1 | 0.8889 |
| **GSE75317_L** | 1(1:0) | 32(1:31) | 0.5 | 1 | 0.6667 |
| **GSE39582_L** | 15(14:1) | 293(4:298) | 0.7778 | 0.9967 | 0.8737 |
| **Total_LCCs** | 23(22:1) | 382(6:376) | 0.7857 | 0.9973 | 0.8789 |
| **GSE13067** | 13(11:2) | 61(0:61) | 1 | 0.9683 | 0.9839 |
| **GSE13294** | 74(70:4) | 81(8:73) | 0.8974 | 0.9481 | 0.9220 |
| **Total_CRCs** | 87(81:6) | 142(8:134) | 0.9101 | 0.9571 | 0.9330 |

Notes: ^a^ represents the predicted MSI status by 10-GPS; ^b^ represent the original MSI status; GSE_L represents the LCC samples; Total_LCCs and Total_CRCs represent all the samples of LCC and CRC, respectively.

**Table S3. The Composition of 6-GPS.**

| **signature** | **gene1** | **gene2** | **signature** | **gene1** | **gene2** |
| --- | --- | --- | --- | --- | --- |
| pair1 | *RTF1* | *FBXO21* | pair4 | *MTA2* | *TNNC2* |
| Pair2 | *ABHD3* | *SEC22B* | pair5 | *LYSMD2* | *STAG2* |
| Pair3 | *LYG1* | *GNL3L* | pair6 | *DDB2* | *TGFBR2* |

Notes:

A LCC sample was classified as MSI if the REOs (gene1>gene2) of at least 4 of the gene pairs in the 6-GPS vote for MSI; otherwise the MSS.

**Table S4. The performances of the 6-GPS in LCCs of the independent datasets.**

|  | **pre-MSI†**  **(MSI:MSS)‡** | **pre-MSS†**  **(MSI:MSS)‡** | **sensitivity** | **specificity** | **F-score** |
| --- | --- | --- | --- | --- | --- |
| **GSE39084_L** | 3(3:0) | 27(0:27) | 1 | 1 | 1 |
| **GSE18088_L** | 5(4:1) | 20(1:19) | 0.8 | 0.95 | 0.8686 |
| **GSE75317_L** | 2(2:0) | 31(0:31) | 1 | 1 | 1 |
| **TCGA_L** | 0(0:0) | 34(1:33) | 0 | 1 | 0 |
| **Total_LCCs** | 10(9:1) | 112(2:110) | 0.8182 | 0.991 | 0.8963 |

Note:

† represents the predicted MSI status by 6-GPS; ‡ represent the original MSI status; GSE_L represents the LCC samples; Total_LCCs represents all the samples of LCC.
