# Supplementary figures and images for: A qualitative transcriptional signature for predicting microsatellite instability status of right-sided Colon Cancer
Source: BMC Genomics. 2019 Oct 23;20:769. doi: 10.1186/s12864-019-6129-8 (PMC6813057; doi:10.1186/s12864-019-6129-8)

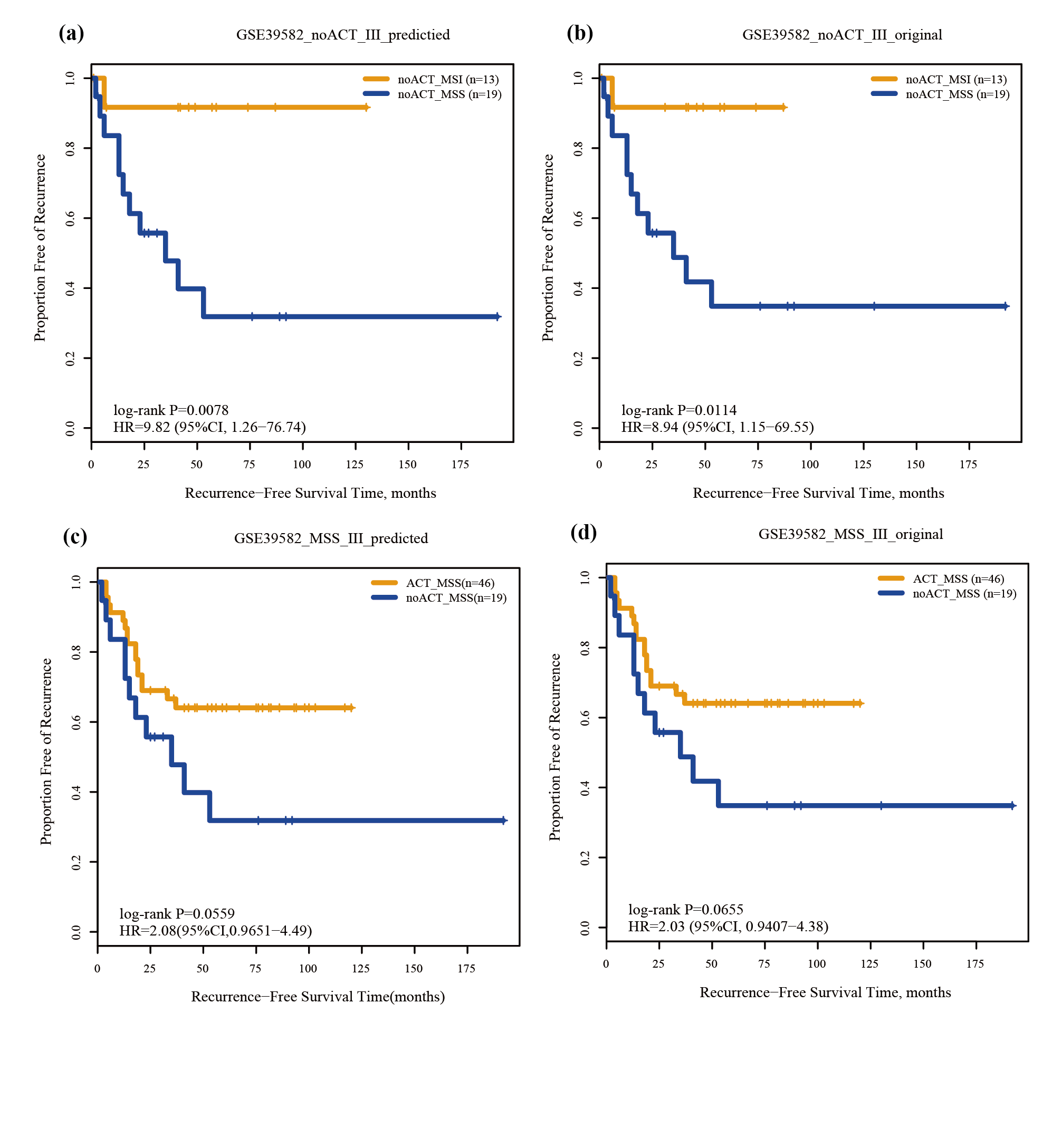

Supplement: Supplementary file 3 — Additional file 3. The Kaplan-Meier survival curve for the prediction of 10-GPS and original MSI status, respectively. (TIF 524 kb) [file 12864_2019_6129_MOESM3_ESM.tif]
